# The application of high-throughput sequencing technology in corneal diseases

**DOI:** 10.1007/s10792-024-03049-1

**Published:** 2024-02-10

**Authors:** Jing yi Zhao, Yu xi He, Mei liang Wu, Rui qing Wang

**Affiliations:** 1 The Second Clinical Medical College of Jilin University, Changchun, 130012 Jilin China; 2 The Eye Center, The Second Hospital of Jilin University, Changchun, 130041 Jilin China

**Keywords:** Corneal diseases, High-throughput sequencing, Disease diagnosis, Adjuvant therapy

## Abstract

High-throughput sequencing technology, also known as next-generation sequencing technology, can explore new biomarkers and specific gene mutations. It has a pivotal role in promoting the gene research, which can limit the detection area, lessen the time needed for sequencing. Also, it can quickly screen out the suspected pathogenic genes of patients, gain the necessary genetic data, and provide the basis for clinical diagnosis and genetic counseling. In the research of corneal diseases, through the DNA sequencing of patients’ diseased cells, it can provide a deeper understanding of corneal diseases and improve the diagnosis, classification and treatment alternatives of various corneal diseases. This article will introduce the application progress of high-throughput sequencing technology in corneal diseases, which will help to understand the application of this technology in various corneal diseases.

## Introduction

In recent years, the incidence rate of corneal diseases is on the rise and corneal diseases have become one of the most common causes of blindness in the world [1]. At present, there are about 4 million blind and disabled people caused by corneal diseases in China. It is expected the amount of blindness because of corneal diseases which are difficult to prevent or treat will continue to increase in the future [2]. Among the corneal diseases, infection, trauma, immune system diseases, genetic diseases, and other corneal diseases would lead to changes in corneal transparency, shape, and integrity. So, it is vital to study the pathogenesis, prevention, and treatment of corneal diseases. Moreover, many corneal diseases possess genetic causes, and some are associated with fairly rare and complex syndromes. In addition, the pathogenesis of these corneal diseases is not clear with the characteristics of occult, heterogeneity, and complexity. On the contrary, it greatly increases the difficulty of early diagnosis, causing some patients to miss the optimal therapeutic time point, and increasing the difficulty of treatment.

With the completion of the Humanity Genome Project, the breakthrough development of high-throughput sequencing technology has promoted the advent of the era of precision medicine [3]. Also, high-throughput sequencing technology opened new ideas for the study of corneal diseases as well as provided effective methods to deal with these difficult problems. Nowadays, high-throughput sequencing technology has gone viral in clinical studies of corneal diseases. Besides, it played a great role in defining the detection area, reducing the time required for sequencing, rapidly screening out the suspected pathogenic genes of patients, obtaining the necessary genetic data, and providing clear clinical diagnosis and genetic counseling. Moreover, it can also offer more therapeutic targets for drugs, and provide a theoretical basis for personalized medicine to achieve an accurate diagnosis and treatment of corneal diseases. So, this paper mainly introduces the application of high-throughput sequencing technology in the clinical diagnosis and treatment of various corneal diseases.

## High-throughput sequencing technology

High-throughput sequencing technology, also known as second-generation sequencing technology, has been continuously developed and perfected in the past decade [4]. With the ability to sequence millions of DNA molecules at a time, it can achieve high throughput, high efficiency, and high accuracy measurement [5], which is why it is also called the next-generation sequencing technology. What’s more, compared with the first-generation sequencing Sanger method, high-throughput sequencing technology has significant advantages in processing large-scale samples and greatly increases sequencing throughput and output, just because of this it becomes a core technology in current genomics research.

**Fig. 1:**
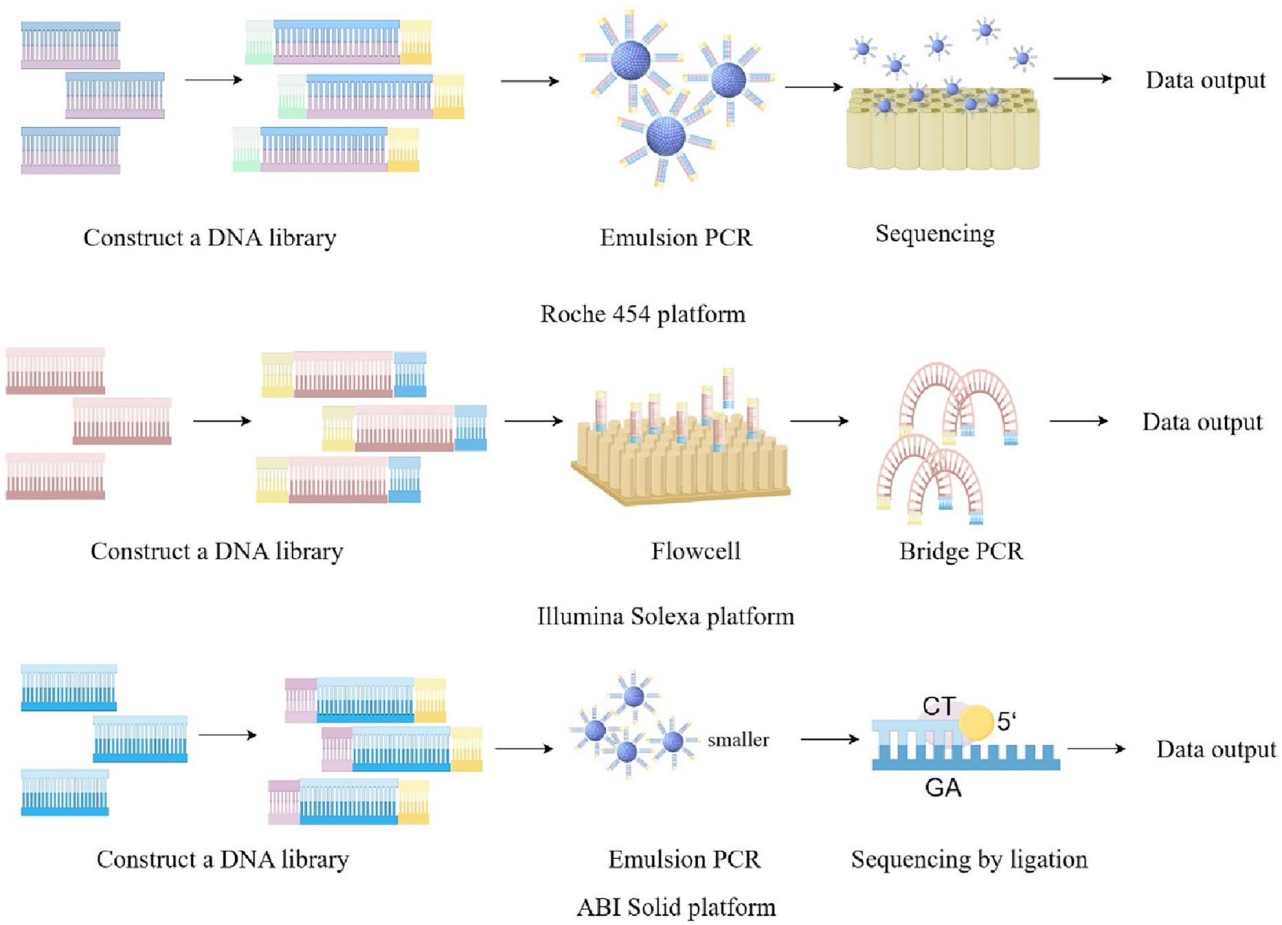
The process of high-throughput sequencing technology (by Figdraw)

Up to now, high-throughput sequencing technology is mainly divided into three platforms: the Roche 454 platform, the Illumina Solexa platform, and ABI Solid platform [6, 7]. Their principles are similar (Fig. 1), but methods in the sequencing process are different: (1) the Roche 454 platform is based on the principle of building a single-stranded DNA library. And the single-stranded DNA is bound to the magnetic beads coated with water droplets for emulsification PCR amplification to reach the amount of DNA required for the next sequencing. The sequencing method uses pyrosequencing, and the sequencing reaction takes single-stranded DNA as a template, for one kind of dNTP adds to each reaction to synthesize the reaction. If dNTP can be paired with the sequence to be sequenced, it would emit a fluorescent signal and obtain the final sequencing result through optical signal processing. The Roche 454 platform can obtain long sequencing read lengths and reduce interference sequencing bias, but it cannot accurately measure homomer length and may introduce insertion and deletion sequencing errors. (2) The core principle of the Illumina Solexa platform is to use ultrasound to break the DNA samples to be tested into small fragments to build a single-stranded DNA library. Flow cell is used to adsorb the channels of flowing DNA fragments, and then bridge amplification is carried out to aggregate the DNA fragments into bundles at their respective positions to realize the amplification of base signal intensity, in order to meet the signal requirements required by sequencing. In general, the sequence is displayed by adding DNA polymerase, coupling primers, and dNTP with specific fluorescent labels, which can solve the problem of accurate measurement of homomer length. (3) The principle of the ABI Solid platform is to break and attach sequencers at both ends of the fragment to connect the vector and construct a single-stranded DNA library for PCR amplification. Emulsion, however, uses ligase instead of DNA polymerase, which is common in DNA sequencing, to determine a fluorescence signal by using two bases. Emulsion Base sequencing at all locations is completed, and bases at each location are detected twice, greatly improving the accuracy of sequencing [8–11].

## Diagnosis of corneal disease

The typical clinical manifestations of corneal diseases include bulbar conjunctival edema, corneal opacity, and corneal neovascularization. Currently, commonly used clinical examination methods include vision examination, slit-lamp biomicroscope for superficial lesions, corneal topographic mapping to analyze the curvature and refractive power of the anterior surface, intraocular pressure measurement, and corneal thickness examination. Additionally, optical coherence tomography (OCT), corneal confocal microscopy, polymerase Chain Reaction (PCR), and sequencing technology have high specificity in diagnosing corneal diseases, each with its own unique advantages and limitations, making them valuable tools for accurate diagnosis (Table 1).

**Table 1:** Accurate diagnosis of corneal diseases

Diagnostic methods	Advantages	Disadvantages	Corneal diseases
OCT [12]	OCT allows for the observation of the cornea’s ultrastructure High resolution and fast imaging	OCT cannot be used for eye diseases that interfere with the passage of light	Keratoconus Corneal dystrophy
Corneal confocal microscopy [13]	Images of tissues and cells from all layers of the living cornea can be obtained High magnification and high resolution	Corneal surface anesthesia is necessary for this examination, and there is a possibility of experiencing symptoms of allergy Due to the contact with the patient's cornea, there is a risk of corneal damage and infection during the examination	Corneal dystrophy Diabetic corneal neuropathy Infections and traumatic lesions
PCR [14]	Testing for known mutations High specificity and high sensitivity	Longer processing time and higher cost It can only detect the presence of specific fragments, and cannot directly determine if a gene has mutated	Infectious corneal diseases Corneal dystrophy Corneal neovascularization
Sequencing technology	The direct determination of the genetic sequence of the lesion's location can also identify unknown mutations	It is necessary to use other auxiliary examination techniques and consider clinical manifestations Prior to sequencing, PCR amplification is required	Inherited corneal diseases Infectious corneal diseases Corneal neovascularization

## Application of high-throughput sequencing technology in corneal diseases

### Application of high-throughput sequencing technology in infectious corneal diseases

#### Bacterial keratitis (BK)

Bacterial keratitis (BK) is the most common type of suppurative keratitis caused by bacterial infection in the corneal epithelium, resulting in necrosis of the corneal stroma beneath the affected area. It usually occurs rapidly and can lead to serious complications such as corneal perforation if it is not treated promptly. Currently, the gold standard for diagnosing BK is germiculture, but its accuracy is limited with a positive rate of only 40–70%. Besides, corneal confocal microscopy can aid in identifying the specific pathogen, but its limited field of view makes it difficult to observe the same location multiple times. Additionally, PCR can amplify the specific bacterial gene sequence with high sensitivity, but it does not have broad spectrum [15].

High-throughput sequencing has the unique advantage of providing taxonomic and functional maps of bacterial communities without the need for culturing microorganisms in the laboratory [16]. This technology accurately defines the classification characteristics of bacteria, improves the diagnosis rate, and allows for comparison between normal and infected bacterial communities. Additionally, high-throughput sequencing can be used to compare with drug resistance databases to obtain information on drug resistance in infected bacteria, allowing for precise treatment. However, high-throughput sequencing still has limitations; for example, it is more expensive and has a longer cycle. Redd TK et al*.* [17] conducted high-throughput metagenomic RNA sequencing on 46 cases of undifferentiated corneal ulcers, and the results showed that high-throughput sequencing has higher specificity and sensitivity than traditional germiculture, making it a valuable assistant diagnostic method for BK. Similarly, Ren et al*.* [18] performed high-throughput 16S rDNA sequencing and germiculture on conjunctivitis swabs and corneal squeezes collected from patients with BK. The results showed that high-throughput 16S rDNA sequencing had a significantly higher positive rate for identifying pathogens causing BK compared to germiculture. Furthermore, the study found that *Actinomyces* and *Coryneomyces* were significantly reduced, while *Pseudomonas*, *Bacteroides*, and *Escherichia Shigella* were more prevalent, suggesting that the imbalance of protective and invasive bacteria in the ocular flora of healthy individuals may contribute to susceptibility to BK. Therefore, regulating the ocular microbiota is a promising new direction for the prevention and treatment of BK.

#### Viral keratitis (VK)

Viral keratitis is a serious blinding corneal disease caused by a viral infection. It can be classified into different types based on the specific virus that causes the infection, such as adenovirus keratitis, herpes simplex keratitis, and herpes zoster keratitis. Symptoms of viral keratitis often include sensitivity to light, tearing, and redness in the eyes. Traditional antiviral therapy (AVP) is not always effective in treating viral keratitis due to factors such as viral drug resistance, inadequate dosage, or poor patient compliance [19]. However, high-throughput sequencing can be used to assess drug resistance in the virus and accurately select appropriate medications, as well as evaluate patient compliance to improve treatment outcomes. Fedaoui et al*.* [20] analyzed the genetic variability of human adenovirus type 8 (HAdV-8) keratoconjunctivitis patients through high-throughput gene sequencing of hexagonal protein, fiber and penton bases, and found that the sequences from HAdV-8 had different mutation characteristics, and the genetic dynamics of possible variation among HAdV-8 isolates. The result is crucial for understanding the genetic characteristics of the adenovirus hypervariable region sequence, identifying the virus genotype, and improving the diagnosis of viral keratitis in combination with clinical symptoms.

#### Fungal keratitis (FK)

Fungal keratitis (FK) is an important blinding corneal disease caused by a pathogenic fungal infection [21]. In recent years, the incidence of the disease has increased significantly. At present, the research on the pathogenesis of fungal infection generally believes that the lack of correct identification of pathogenic fungi is the key and difficulty in diagnosis and treatment.

Except conventional diagnostic methods such as fungal culture, PCR has become an important tool in the diagnosis of FK. PCR involves amplifying fungal DNA sequences obtained from keratopathy samples in order to identify the specific fungi. It is rapid and highly sensitive, making it particularly useful for detecting early infections with low fungal load. However, it is important to note that while panfungal primers can be used to identify the presence of fungal DNA, specific primers are needed to identify specific fungi. Additionally, the PCR process may lead to cross-contamination with pathogenic flora, potentially resulting in overdiagnosis [22]. Another emerging method for early diagnosis of FK is in vivo confocal microscopy (IVCM). It allows for dynamic observation of fungal mycelium morphology in the cornea and is widely used in ocular fungal infections. IVCM also has the ability to detect the depth of fungal infection with high sensitivity and specificity. However, IVCM is unable to distinguish between *Escherichia Shigella* [23].

High-throughput sequencing can obtain specific fungal sequences, so as to make a clear classification diagnosis, and it also monitors the dynamic changes of fungal flora related to FK, providing a basis for the clinical course of FK at different stages and the formulation of treatment strategies. Ren et al*.* [24] performed ITS sequencing on the DNA extracted from corneal cells of 38 patients who were highly suspected of FK. The results showed that compared with fungal culture, ITS sequencing could directly generate the ITS sequence of the fungus and obtain detailed information about the fungus, with high sensitivity and specificity. B00MIRAJH plays a potential role in regulating pathogenesis as a gene that can be predicted to specifically target wound inflammation. Boomiraj et al*.* [25] combined normal donor corneas and corneas of fungal keratitis patients, respectively, and high-throughput miRNAs showed that highly dysregulated miRNAs (Mir-511-5p, Mir-142-3p, Mir-155-5p and Mir-451a) were closely related to wound healing. Therefore, the results of high-throughput sequencing technology further found the important role of key genes in the occurrence of fungal keratitis and provided new ideas for the diagnosis of fungal keratitis.

### Application of high-throughput sequencing technology in inherited corneal diseases

#### Corneal dystrophy

Corneal dystrophy is a progressive keratopathy that is primarily inherited within families. Abnormal genes regulate cells in normal corneal tissue, leading to gradual damage to the structure and function of the cornea. According to the location of the disease, it can be divided into the following three categories: (1) Anterior corneal dystrophy, involving the epithelium and Bowman membrane; (2) Stromal corneal dystrophy involving the stroma or central layer of the cornea; (3) Posterior corneal dystrophy involving Descemet membranes and endodermis. Corneal dystrophy is typically inherited in an autosomal pattern. Due to the specific pathogenic gene and chromosome localization of different subtypes, patients with the same gene mutation may exhibit varying clinical manifestations (Table 2).

**Table 2:** The classification of corneal dystrophy

Classification	Type	Pathogenic genes	Chromosomal mapping	Inheritance patterns
Anterior corneal dystrophy	Epithelial basement membrane dystrophy	TGFBI	5q31.1	AD
	Meesmann corneal dystrophy	KRT3	12q13.13	AD
		KR12	17q21.2	
	Reis-Bucklers corneal dystrophy	TGFBI	5q31.1	AD
	Thiel-Behnke corneal dystrophy	TGFBI	5q31.1	AD
Corneal stromal dystrophy	Gelatinous drop-like corneal dystrophy	TACSTD2	1p32.1	AR
	Granular corneal dystrophy	TGFBI	5q31.1	AD
	Lattice corneal dystrophy type I/III	TGFBI	5q31.1	AD
		TACSTD2	1p32.1	AR
	Macular corneal dystrophy	CHST6	16q23.1	AR
	Schnyder corneal dystrophy	UBIAD1	1p36.22	AD
Posterior corneal dystrophy	CHED	SLC4A11	20p13	AR
	FECD I/II/III	COL8A2	1p34.3	AD
		FECD2	13pter-q12.13	AD
		TCF4	18q21.2	AD
	Posterior polymorphous corneal dystrophy	OVOL2	20p11.23	AD
		COL8A2	1p34.3	AD

At present, the clinical diagnosis of corneal dystrophy needs to be combined with a detailed eye examination, including the examination of the anterior chamber Angle, lens and posterior segment of the eye. Imaging techniques, such as photography, red reflection photographs, optical coherence tomography, mirror microscopy, and living corneal confocal microscopy can be used to provide a comprehensive view of the condition [26].

However, a definitive diagnosis requires genetic testing. High-throughput sequencing was performed on genes associated with corneal dystrophy to screen out mutant genes, supplemented by molecular karyotype analysis in order to clarify the typing and genetic pattern, and thus achieve precise treatment. Riazuddin et al*.* [27] used high-throughput sequencing technology to carry out whole gene scanning and found that missense mutation C.2969 g > C in AGBL1. This mutation affects the encoding of AGBL1, a cytoplasmic glutamate decarboxylase, and reduces its interaction with TCF4. The authors suggest that TCF4 may be an enzymatic target of AGBL1 and this interaction could potentially explain the development of Fuchs endothelial corneal dystrophy (FECD). Zhang et al*.* [28] performed TRIa-based whole exome sequencing on a patient with congenital hereditary endothelial dystrophy (CHED) and their parents. They identified a homozygous mutation in the FAM149A gene, which is highly expressed in corneal endothelial cells and up-regulated during oxidative stress. This finding supports the role of FAM149A as a pathogenic gene in the development of CHED and provides new insights for future studies. Gong et al*.* [29] extracted intracellular mRNA from the peripheral blood of a lineage of maculopathy corneal dystrophy, generated cDNA by reverse transcription, synthesized specific primers, and designed primer sequences. They amplified all exons of the target gene by polymerase chain reaction technology and performed high-throughput sequencing to compare with the normal population. 1072 T > C(Y358H) was found to be a CHST6 gene mutation [30]. This study is helpful to clarify the pathogenesis of this disease and provide a basis for targeted gene therapy.

In summary, high-throughput sequencing technology can accurately locate gene mutations and clarify the types and genetic patterns of corneal dystrophy, providing a basis for targeted gene therapy. However, it also has limitations and should be used in conjunction with clinical manifestations and ophthalmic examination for an accurate diagnosis of corneal dystrophy. It cannot be solely relied upon as an auxiliary tool in clinical settings.

#### Keratoconus (KC) pathogenesis

Keratoconus (KTCN) [31] is a corneal disease characterized by corneal dilatation, thinning of the central corneal stroma, and corneal cones. At present, the pathogenesis of the disease is not clear, and it is the result of the joint action of many factors [32].

Magalhaes et al*.* [33] performed high-throughput whole exome sequencing in 6 keratoconus patients. A heterozygous missense mutation c.4678C > T was identified by intra-family co-isolation to verify the pathogenic mutation. Rabinowitz et al. [34] high-throughput whole exome sequencing of microRNAs expressed in the cornea and lens confirmed to some extent that microRNA 184 plays an important role in the development of keratoconus. It can be seen that high-throughput sequencing can quickly find and identify the mutation sites of the pathogenic genes, which helps researchers to analyze the relationship between genes and phenotypes, and provides a good condition for exploring the possible genetic factors between keratoconus patients and their first-degree relatives.

In addition to the numerous sequencing studies focused on genomic DNA, a few studies have also been conducted on the level of mitochondrial DNA to identify further genetic aspects of KTCN [35]. An investigation of complete mitochondrial DNA taken from blood samples taken from KTCN patients in Saudi Arabia revealed that 38.5% of KTCN patients had mitochondrial DNA variants. Of the identified variations, only one non-synonymous variant (m.4218 T > A in ND1) was heterogeneous, while the remaining nine were homologous [36]. Besides, analysis of mitochondrial DNA gives insight into oxidative damage in KTCN, which will play an important role in the further study of pathogenesis.

#### Brittle cornea syndrome (BCS)

Brittle corneal syndrome (BCS) [37] is an autosomal recessive disease characterized by extreme corneal thinning and fragility, leading to frequent corneal perforations. Other ocular features included blue sclera, corneal astigmatism, and high myopia. In addition, BCS is also a genetically heterogeneous disease: two allelic mutations of the ZNF469 gene on chromosome 16q24 and the PRDM5 gene on chromosome 4q27 are all related to the disease [38].

In order to quickly and easily obtain the gene mutation spectrum of the patient, Rolvien et al*.* [39] performed high-throughput sequencing on the sister with BCS in childhood, detected the ZNF469 finding variant and reexamined the patient to determine other typical features of BCS. Agnes Selina et al*.* [40] performed high-throughput exome sequencing on blood samples collected from children with clinical features of BCS syndrome type II and their brothers and parents. To find patterns of inheritance, they performed pedigree analysis and internal validation. Finally, a homozygous shift variant and a mutant SLC6A5 gene were identified in exon 6 of the PRDM5 gene, and this novel combination of PRDM5 and SLC6A5 mutations were thought to be responsible for the SLC6A5 mutation. In summary, high-throughput sequencing technology can clarify the genotype diagnosis of patients, and provide a basis for further diagnosis and treatment options, thereby expanding the scope of clinical diagnosis and treatment of genetic diseases.

### Application of high-throughput sequencing technology in idiopathic corneal diseases

#### Corneal neovascularization

The cornea is normally an avascular, clear connective tissue. Corneal neovascularization occurs in pathological conditions caused by inflammation such as burns and ischemia, which can lead to blindness in severe cases [41]. The pathogenesis of corneal neovascularization is still unclear. It is generally believed that it is related to the abnormal corneal limbal anatomy and function, the increase of pro-angiogenic factor, the decrease of inhibitory angiogenic factor, the immune inflammatory reaction, corneal edema and hypoxia. Corneal neovascularization can be induced by many ocular immunoinflammatory, infectious, degenerative, traumatic and iatrogenic diseases. Studies have shown that miRNA can regulate angiogenesis by regulating cell migration, which is closely related to corneal neovascularization. Among them, the application of high-throughput sequencing technology to analyze and determine the miRNA related to corneal inflammation and angiogenesis can be analyzed and identified for specific therapeutic intervention [42].

## Conclusions and outlook

In recent years, the concept of “precision medicine” has gained traction, making high-throughput sequencing technology an essential tool. In the field of genetics, high-throughput sequencing has greatly advanced the research of genes. By limiting the detection area and shortening the sequencing time, it can quickly screen out the suspected pathogenic genes of patients and then obtain the necessary genetic data to provide the necessary data support for a clear clinical diagnosis. Furthermore, high-throughput sequencing can systematically reveal the genetic patterns underlying corneal diseases, providing a theoretical basis and data support for further research.

With the emergence of artificial intelligence, the development of deep learning systems offers a new approach to diagnosing corneal diseases. Therefore, the combination of high-throughput sequencing and artificial intelligence is expected to be the future trend in accurately diagnosing corneal diseases. By utilizing high-throughput sequencing and deep learning, it is possible to achieve high-precision detection of corneal diseases through non-invasive corneal images and automatic analysis of corneal cell components and infectious microorganisms. This not only addresses the limitations of high-throughput sequencing, such as long processing times, but also enables accurate classification and diagnosis of various corneal diseases. The integration of high-throughput sequencing technology and artificial intelligence is a promising area for the future development.

## References

[1] Di Iorio E, Barbaro V, Alvisi G et al (2019) New frontiers of corneal gene therapy. Hum Gene Ther 30(8):923–925 31020856 10.1089/hum.2019.026

[2] Brunette I, Roberts CJ, Vidal F et al (2017) Alternatives to eye bank native tissue for corneal stromal replacement. Prog Retin Eye Res 59:97–130 28450146 10.1016/j.preteyeres.2017.04.002

[3] Morganti S, Tarantino P, Ferraro E et al (2019) Complexity of genome sequencing and reporting: next generation sequencing (NGS) technologies and implementation of precision medicine in real life. Crit Rev Oncol Hematol 133:171–182 30661654 10.1016/j.critrevonc.2018.11.008

[4] Sabour L, Sabour M, Ghorbian S (2017) Clinical applications of next-generation sequencing in cancer diagnosis. Pathol Oncol Res 23(2):225–234 27722982 10.1007/s12253-016-0124-z

[5] Hu T, Chitnis N, Monos D et al (2021) Next-generation sequencing technologies: an overview. Hum Immunol 82(11):801–811 33745759 10.1016/j.humimm.2021.02.012

[6] Slatko BE, Gardner AF, Ausubel FM (2018) Overview of next-generation sequencing technologies. Curr Protoc Mol Biol 122(1):e59 29851291 10.1002/cpmb.59 PMC6020069

[7] Van Dijk EL, Auger H, Jaszczyszyn Y et al (2014) Ten years of next-generation sequencing technology. Trends Genet 30(9):418–426 25108476 10.1016/j.tig.2014.07.001

[8] Forde BM, O’Toole PW (2013) Next-generation sequencing technologies and their impact on microbial genomics. Brief Funct Genom 12(5):440–453 10.1093/bfgp/els062 23314033

[9] Kamps R, Brandão RD, Bosch BJ et al (2017) Next-generation sequencing in oncology: genetic diagnosis, risk prediction and cancer classification. Int J Mol Sci 18(2):308 28146134 10.3390/ijms18020308 PMC5343844

[10] Reuter JA, Spacek DV, Snyder MP (2015) High-throughput sequencing technologies. Mol Cell 58(4):586–597 26000844 10.1016/j.molcel.2015.05.004 PMC4494749

[11] Kircher M, Kelso J (2010) High-throughput DNA sequencing–concepts and limitations. BioEssays 32(6):524–536 20486139 10.1002/bies.200900181

[12] Ang M, Baskaran M, Werkmeister RM et al (2018) Anterior segment optical coherence tomography. Prog Retin Eye Res 66:132–156 29635068 10.1016/j.preteyeres.2018.04.002

[13] Petropoulos IN, Ponirakis G, Khan A et al (2020) Corneal confocal microscopy: ready for prime time. Clin Exp Optom 103(3):265–277 30834591 10.1111/cxo.12887

[14] Yera H, Ok V, Lee Koy Kuet F et al (2021) PCR and culture for diagnosis of Acanthamoeba keratitis. Br J Ophthalmol 105(9):1302–1306 33099504 10.1136/bjophthalmol-2020-316730

[15] Borroni D, Bonzano C, Sánchez-González JM et al (2023) Shotgun metagenomic sequencing in culture negative microbial keratitis. Eur J Ophthalmol 33(4):1589–1595 36617769 10.1177/11206721221149077

[16] Borroni D (2022) Granulicatella adiacens as an unusual cause of microbial keratitis: a metagenomic approach. Ocul Immunol Inflamm 30(6):1550–1551 34236294 10.1080/09273948.2021.1933066

[17] Redd TK, Lalitha P, Prajna NV et al (2022) Impact of sample collection order on the diagnostic performance of metagenomic deep sequencing for infectious keratitis. Cornea 41(1):39–44 34870622 10.1097/ICO.0000000000002766 PMC8649208

[18] Ren Z, Liu Q, Li W et al (2021) Profiling of diagnostic information of and latent susceptibility to bacterial keratitis from the perspective of ocular bacterial microbiota. Front Cell Infect Microbiol 11:645907 34055665 10.3389/fcimb.2021.645907 PMC8155582

[19] Rousseau A, Boutolleau D, Titier K et al (2017) Recurrent herpetic keratitis despite antiviral prophylaxis: a virological and pharmacological study. Antiviral Res 146:205–212 28939476 10.1016/j.antiviral.2017.09.013

[20] Fedaoui N, Ayed NB, Yahia AB et al (2016) Genetic variability of human adenovirus type 8 causing epidemic and sporadic cases of keratoconjunctivitis. Arch Viro 161(6):1469–1476 10.1007/s00705-016-2804-0 26957298

[21] Maharana PK, Sharma N, Nagpal R et al (2016) Recent advances in diagnosis and management of mycotic keratitis. Indian J Ophthalmol 64:346–357 27380973 10.4103/0301-4738.185592 PMC4966371

[22] Sharma S, Rathi VM, Murthy SI et al (2021) Application of trypan blue stain in the microbiological diagnosis of infectious keratitis-a case series. Cornea 40(12):1624–1628 33935235 10.1097/ICO.0000000000002725

[23] Chidambaram JD, Prajna NV, Larke N et al (2017) In vivo confocal microscopy appearance of *Fusarium* and *Aspergillus* species in fungal keratitis. Br J Ophthalmol 101(8):1119–1123 28043985 10.1136/bjophthalmol-2016-309656 PMC5537506

[24] Ren Z, Liu Q, Wang Y et al (2020) Diagnostic information profiling and evaluation of causative fungi of fungal keratitis using high-throughput internal transcribed spacer sequencing. Sci Rep 10(1):1640 32015395 10.1038/s41598-020-58245-7 PMC6997210

[25] Boomiraj H, Mohankumar V, Lalitha P (2015) Human corneal microRNA expression profile in fungal keratitis. Invest Ophthalmol Vis Sci 56(13):7939–7946 26720440 10.1167/iovs.15-17619

[26] Weiss JS, Willoughby CE, Abad-Morales V et al (2022) Update on the corneal dystrophies-genetic testing and therapy. Cornea 41(11):1337–1344 36219210 10.1097/ICO.0000000000002857

[27] Riazuddin SA, Zaghloul NA, Al-Saif A et al (2010) Missense mutations in TCF8 cause late-onset fuchs corneal dystrophy and interact with FCD4 on chromosome 9p. Am J Hum Genet 86(1):45–53 20036349 10.1016/j.ajhg.2009.12.001 PMC2801746

[28] Zhang J, Dai Y, Wu D et al (2021) Whole exome sequencing identified FAM149A as a plausible causative gene for congenital hereditary endothelial dystrophy, affecting Nrf2-Antioxidant signaling upon oxidative stress. Free Radic Biol Med 173:117–124 34303830 10.1016/j.freeradbiomed.2021.07.029

[29] Gong M, Zhao X, Zhang M (2015) Study on new CHST6 mutation in consanguineous families with macular corneal dystrophy. New prog ophthalmol 12(35):1141–1144

[30] Tan DT, Dart JK, Holland EJ et al (2012) Corneal transplantation. Lancet 379(9827):1749–1761 22559901 10.1016/S0140-6736(12)60437-1

[31] Mas Tur V, MacGregor C, Jayaswal R et al (2017) A review of keratoconus: diagnosis, pathophysiology, and genetics. Surv Ophthalmol 62(6):770–783 28688894 10.1016/j.survophthal.2017.06.009

[32] Hashemi H, Heydarian S, Hooshmand E et al (2020) The prevalence and risk factors for keratoconus: a systematic review and meta-analysis. Cornea 39(2):263–270 31498247 10.1097/ICO.0000000000002150

[33] Magalhães OA, Kowalski TW, Wachholz GE et al (2019) Whole-exome sequencing in familial keratoconus: the challenges of a genetically complex disorder. Arq Bras Oftalmol 82(6):453–539 31482965 10.5935/0004-2749.20190087

[34] Rabinowitz YS, Galvis V, Tello A et al (2021) Genetics vs chronic corneal mechanical trauma in the etiology of keratoconus. Exp Eye Res 202:108328 33172608 10.1016/j.exer.2020.108328

[35] Karolak JA, Gajecka M (2017) Genomic strategies to understand causes of keratoconus. Mol Genet Genom 292(2):251–269 10.1007/s00438-016-1283-z PMC5357269 28032277

[36] Abu-Amero KK, Azad TA, Kalantan H et al (2014) Mitochondrial sequence changes in keratoconus patients. Invest Ophthalmol Vis Sci 55(3):1706–1710 24569587 10.1167/iovs.14-13938

[37] Cundy T, Vincent A, Robertson S (2021) Does brittle cornea syndrome have a bone fragility phenotype? Bone Rep 15:101124 34522702 10.1016/j.bonr.2021.101124 PMC8426531

[38] Micheal S, Siddiqui SN, Zafar SN et al (2019) Identification of a novel ZNF469 mutation in a Pakistani family with brittle cornea syndrome. Cornea 38(6):718–722 30865045 10.1097/ICO.0000000000001828

[39] Rolvien T, Kornak U, Linke SJ et al (2020) Whole-exome sequencing identifies novel compound heterozygous znf469 mutations in two siblings with mild brittle cornea syndrome. Calcif Tissue Int 107(3):294–299 32671420 10.1007/s00223-020-00721-3 PMC7415034

[40] Selina A, John D, Loganathan L et al (2020) Case report of a PRDM5 linked brittle cornea syndrome type 2 in association with a novel SLC6A5 mutation. Indian J Ophthalmol 68(11):2545–2547 33120686 10.4103/ijo.IJO_325_20 PMC7774228

[41] Torricelli AA, Santhanam A, Wu J et al (2016) The corneal fibrosis response to epithelial-stromal injury. Exp Eye Res 142:110–118 26675407 10.1016/j.exer.2014.09.012 PMC4683352

[42] Mukwaya A, Jensen L, Peebo B et al (2019) MicroRNAs in the cornea: role and implications for treatment of corneal neovascularization. Ocul Surf 17(3):400–411 30959113 10.1016/j.jtos.2019.04.002

